# Supplementary material for: Characterization of Cortical and Subcortical Structural Brain Asymmetry in Adults with and without Dyslexia
Source: Brain Sci. 2023 Nov 23;13(12):1622. doi: 10.3390/brainsci13121622 (PMC10741947; doi:10.3390/brainsci13121622)
Supplement: Supplementary file 1 [file brainsci-13-01622-s001.zip › brainsci-2674834-supplementary.pdf]

Supplementary Material

Table S1. Between Scanner Independent Samples t-tests.

	**Mean Difference**	**p-value**
Age	1.3	0.106
TOWRE Real Words	0.57	0.971
TOWRE Nonwords	2.9	0.387
Planum Temporale (Total)	0.19	0.378
Fusiform (Total)	0.47	0.466
Supramarginal Gyrus (Total)	1.11	0.068
Caudate (Total)	0.43	0.067
Putamen (Total)	0.22	0.340
Thalamus (Total)	0.97	<0.001*
Caudate (Asymmetry)	1.23	0.077
Putamen (Asymmetry)	0.81	0.158
Thalamus (Asymmetry)	2.82	<0.001*
Supramarginal Gyrus (Asymmetry)	4.26	0.073
Fusiform (Asymmetry)	0.65	0.809
Planum Temporale (Asymmetry)	4.92	0.359

*Follow-up independent t-tests found no significant differences between skilled and impaired readers on Thalamus Total or Thalamus Asymmetry, for the 1.5 T (0.067 and 0.138, respectively)scanner and the 3.0 T scanner (0.567 and 0.821, respectively).
